# Space Electroosmotic Thrusters in Ion Partitioning Soft Nanochannels

**DOI:** 10.3390/mi12070777

**Published:** 2021-06-30

**Authors:** Jiaxuan Zheng, Yongjun Jian

**Affiliations:** School of Mathematical Science, Inner Mongolia University, Hohhot 010021, China; 31536015@mail.imu.edu.cn

**Keywords:** electroosmotic thrusters (EOTs), soft nanochannel, ion partitioning effect, polyelectrolyte layer (PEL)

## Abstract

Space electroosmotic thrusters (EOTs) are theoretically investigated in a soft charged nanochannel with a dense polyelectrolyte layer (PEL), which is considered to be more realistic than a low-density PEL. When the PEL is dense, its permittivity is smaller than the one of the electrolyte solution layer, leading to rearrangement of ions in the channel, which is denoted as the ion partitioning effect. It is noted that fluid viscosity becomes high within the PEL owing to the hydration effect. An analytical solution for electroosmotic velocity through the channel is obtained by utilizing the Debye–Hückel linearization assumption. Based on the fluid motion, thruster performances, including thrust, specific impulse, thrust-to-power ratio, and efficiency, are calculated. The ion partitioning effect leads to enhancement of the thruster velocity, while increase of the dynamic viscosity inside the PEL reduces the flow rate of the fluid. Therefore, these performances are further impacted by the dense soft material, which are discussed in detail. Moreover, changes or improvements of the thruster performances from the dense PEL to the weak PEL are presented and compared, and distributions of various energy items are also provided in this study. There is a good result whereby the increase in electric double layer thickness promotes the development of thruster performances. Ultimately, the simulated EOTs produce thrust of about 0 to 20 μN and achieve thruster efficiency of 90.40%, while maintaining an appropriate thrust–power ratio of about 1.53 mN/W by optimizing all design parameters.

## 1. Introduction

In the last two decades, micro/nano-spacecrafts have attracted considerable attention owing to their inherent advantages, such as decreasing launch costs, improving accuracy and efficiency, as well as dispersing space mission risks, and so on [1,2]. Miniaturization of space components has promoted the development of micro/nano-thrusters in the space propulsion system, where the electrokinetic propulsion technology becomes a new solution of space-related tasks [3,4,5,6,7,8]. Motivated by the electrokinetic electroosmosis principle, electroosmosis actuation with an external electric field is extensively applied to micro/nano-fluidics and electric machines, such as micropump devices and small thrusters, which are associated with the electric double layer (EDL) in micro/nanochannels [9,10,11]. The EDL is generated due to the interaction of ionized solution with static charges on dielectric surfaces. When an external electric field is employed along the solid surface, the mobile ions in the EDL are actuated by the electrostatic body force so that the adjacent liquid layer is dragged via the fluid viscous effect. This is just the classical electroosmotic flow (EOF) [12]. The EOF has been extensively investigated and discussed by many experts and scholars in micro/nanochannels [13,14,15,16], which is feasible to be applied to the space propulsion system. Dize et al. [17] theoretically investigated the performances of nano-electrokinetic thrusters driven by electroosmosis forces, which achieved high thruster efficiency with corresponding high thrust-to-power ratios. Huang and Huang [18] spontaneously extended the two-layer fluidic electroosmotic thrusters and obtained analytical solutions for the flow velocity and thruster performances in slit microchannels.

The aforementioned studies about the electroosmotic thrusters were related to hard/rigid microchannels, so the EOTs with soft material walls can be further considered. The electrokinetic characteristics of soft materials have been focused on and widely implemented in micro/nano-systems [19,20,21,22,23,24]. Bartolo and Aarts [25] showed the advantage of the microfluidic systems combining soft matter with biophysics. Hu and Wu [26] established the mathematical model of electroosmotic consolidation with soft materials. In this article, we theoretically investigate and analyze performances of the EOTs in soft nanochannels with dense polyelectrolyte walls for space electroosmotic propulsion. Unlike the hard/rigid channel, the soft channel includes a charged polyelectrolyte layer (PEL), which is also known as a fixed charge layer (FCL). The Donnan potential and additional drag force within the FCL affect the DEL electrostatic potential and electroosmotic velocity in the entire channel, so that the thruster performances are further altered by them. The effect of soft material channels on the electroosmotic flow and electric double layer has been investigated and studied in the past decades [27,28,29,30,31,32,33]. The active controls of the Donnan potential and electrokinetic flow could be regulated, as by Benson et al. [34], in a soft nanochannel with a polyelectrolyte brush layer. Recently, Zheng and Jian [35] executed the space electroosmotic thrusters (EOTs) in a soft nanochannel, but they only considered weak grafting density of the polyelectrolyte layer, which offers a special situation of identical permittivity and viscosity both outside and inside the PEL. Hence, the effect of dense polyelectrolyte materials on space electroosmotic thrusters is focused on in this article, so that model results can be closer to reality than those of previous research.

When the PEL is dense, there is a different tendency of ions to accumulate between within and outside the polyelectrolyte layer so that its permittivity is smaller than the one of the electrolyte solution layer, which leads to major variations in the ionic concentration distribution. Under such a circumstance, the ions are rearranged in the soft channel, which is called the ion partitioning effect [36,37]. Barbosa et al. [38] analyzed the ion partition and membrane potential using the Poisson–Boltzmann equation and observed concentration of ions inside the cell. Reshadi and Saidi [39] accounted for the role of partitioning of finite sized ions in electrohydrodynamic characteristics, considering the different permittivity for the two layers. It is worth nothing that the fluid viscosities of the electrolyte solution layer and PEL are different for high grafting densities owing to hydration effects [40,41,42]. The ion partitioning and hydration effects significantly impact the electrostatics of soft surfaces and the electrokinetic phenomena inside soft channels [43]. Functionalized micro/nanochannels with dense polyelectrolyte layers were reviewed by Ashrafizadeh et al. [44,45], which could realize preferable electrostatic characteristics of the working fluid. The nanochannel arrays with densely charged polyelectrolyte material are devised by Kwak et al. [46] to acquire the generation of power. Hence, electrokinetic properties and energy distributions of propellant in EOTs are affected by the dense polyelectrolyte layers, which are characterized by some parameters such as permittivity, viscosity, EDL thickness, and so on. Controlling and analyzing these correlative parameters are necessary to achieve high thruster efficiency and low power consumption.

In this article, we theoretically investigate the performances of the space electroosmotic thrusters through a soft nanochannel with the dense polyelectrolyte layer. The research is an extension of soft channel influences on electroosmotic thrusters [35], and mainly presents some changes or improvements of EOT performances in ion partitioning soft nanochannels. That is to say, this is an issue about the soft channel of the thruster from the weak PEL to the dense PEL. We want not only to maintain the high energy efficiency of electroosmotic thrusters, but also further to know practical features of the soft material wall in nano-thrusters. Based on the Debye–Hückel linearization assumption, analytical solutions of the fluid electric potential and flow velocity through the soft nanochannel are obtained by the method of dimensionless transformation to simplify the Poisson–Boltzmann (P-B) equation and Naiver–Stokes (N-S) equation, considering different permittivity and viscosity of the two layers. Hence, the thruster performances generated by the fluid motion, including the thruster thrust, specific impulse, efficiency, and thrust-to-power ratio, can be calculated and analyzed, which are affected by the ion partitioning effect, different viscosity, as well as slip length and EDL thickness. Kinetic energy and energy dissipation items containing Joule heating, viscous dissipation, as well as wall sliding friction are presented and discussed in this research. Compared to the electroosmotic thrusters with the walls of low grafting density polyelectrolyte material [35], electroosmotic thrusters (EOTs) of the present study better show the thrust capacity and thruster efficiency in the soft channel at high grafting densities, meanwhile maintaining a not-bad thrust-to-power ratio.

## 2. Mathematical Model Analysis

**Figure 1:**
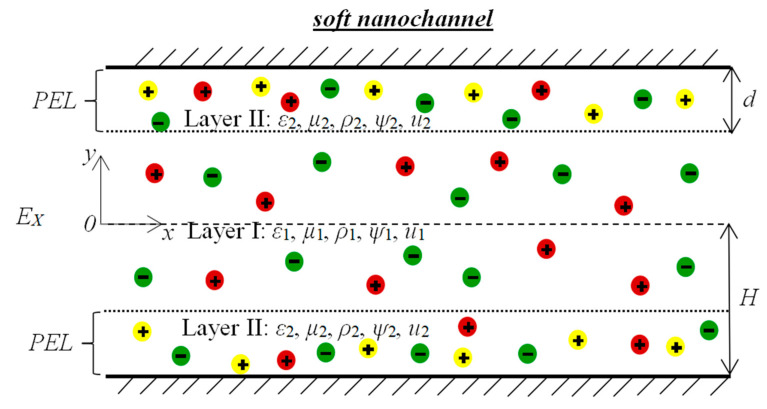
Schematic diagram of the soft nanochannel.

The soft nanochannel of electroosmotic thrusters is shown in Figure 1. The working fluid between two parallel plates or slit-like channels is deemed as an incompressible viscous Newtonian fluid. The fluid is driven by electroosmotic body forces, which are generated by the applied electric field at strength *Ex* along the main flow direction, and synchronously squirted from the channel to obtain the required thrust and energy. The nanochannel height is defined as 2*H*, the length is *L*, and the depth is *W*. It is assumed that the length and depth are both much larger than the height, i.e., *L >> H, W >> H*, so that the flow field is simplified as unidirectional and inertia-free. The soft channel includes a wall-grafted, ion-penetrable, charged polyelectrolyte layer (PEL) whose thickness is set as *d*. The PEL is considered to be homogenous and dense so that the rearrangement of ions reacts and Born energy appears in the entailing electrokinetic system. Physically speaking, the Born energy means the work of diverting an ion from the bulk solvent to the bulk of the membrane, which is related to the fluid permittivity. Different permittivity and fluid viscosities for the electrolyte layer and PEL are considered so as to acquire valid solutions at high PEL densities. The electrolyte solution layer and the dense PEL are viewed as layer I and layer II respectively, with layer I sandwiched by layer II, as shown in Figure 1. We further establish a two-dimensional Cartesian coordinate system at the channel center for convenience. It is hypothesized that the liquid is an ideal steady, fully developed salt that is symmetric in regard to the *x*-axis, and the temperature over the channel cross-section is negligible. Under these conditions, the fluid electric potential and charge density are calculated at an average temperature.

### 2.1. Electric Potential

Due to the symmetry condition, governing equations are described on the upper half region of the nanochannel, i.e., 0 ≤ *y ≤ H*. In order to investigate EOT performances in the soft nanochannel with a dense PEL, we firstly understand electrostatic potential distribution based on the principle of ion partitioning. The electric potential of the two layers, which satisfies the one-dimensional Poisson distribution, is written as:

(1) $$\frac{d^{2}\psi_{1}}{dy^{2}}=-\frac{\rho_{e1}}{\varepsilon_{1}\varepsilon_{0}},0\leq y\leq H-d$$

(2) $$\frac{d^{2}\psi_{2}}{dy^{2}}=-\frac{\rho_{e2}}{\varepsilon_{2}\varepsilon_{0}}-\frac{eNZ}{\varepsilon_{2}\varepsilon_{0}},H-d\leq y\leq H$$

in which *ψ*_1_(*y*) and *ψ*_2_(*y*) represent the electrical potentials of the electrolyte solution layer and polyelectrolyte layer, namely layer I and layer II, *ε*_1_ and *ε*_2_ are the relative permittivities of layer I and layer II, and *ε*_0_ is the permittivity of a vacuum. *e* stands for the proton charge, and *Z* and *N* are the valence and the ionic number concentration of the PEL ions. The above equations indicate that the solution ions exist both within and outside the PEL considering its permeable nature, while the PEL ions only present within the PEL. Besides, *ρ*_*e*1_ and *ρ*_*e*2_ are the volume charge densities for the two layers. They obey the Boltzmann distribution:

(3) $$\rho_{e1}=ez{\left(n_{+}-n_{-}\right)}_{1}$$

(4) $$\rho_{e2}=ez{\left(n_{+}-n_{-}\right)}_{2}$$

where *z* is the valence number of the electrolyte ions, and *n_+_* and *n_−_* are the ionic number concentrations of cations and anions, respectively. In the presence of ion partitioning effects, the number density of the electrolyte ions obeys the modified Boltzmann equation, namely:

(5) $$n_{\pm }=n_{0}\exp(\mp \frac{ze}{k_{b}T_{av}}\psi ),0\leq y\leq H-d$$

(6) $$n_{\pm }=n_{0}\exp(\mp \frac{ze}{k_{b}T_{av}}\psi )\exp(-\frac{\Delta W_{\pm }}{k_{b}T_{av}}),H-d\leq y\leq H$$

where the bulk number concentration *n*_0_ stands for the ionic number concentration of cations and anions in the bulk electrolyte reservoir (where *ψ* = 0) connecting the soft nanochannel, *k_b_* is the Boltzmann constant, and *T_av_* is the average temperature. It is worth nothing that Equations (5) and (6) remain valid for different EDL thicknesses, so the Boltzmann distribution is able to be used even though EDLs overlap [47,48,49]. The term ∆*W_±_*, the Born energy difference, identifies the ion partitioning effect, which is related to different permittivities for the two layers. It is given as:

(7) $$\Delta W_{\pm }=\frac{e^{2}z^{2}}{8\pi r_{\pm }}(\frac{1}{\varepsilon_{0}\varepsilon_{2}}-\frac{1}{\varepsilon_{0}\varepsilon_{1}})$$

in which *r_±_* is the hydrated radius of ionic species. In the symmetric salt solution, for instance KCL solution, the hydrated radii of ions is assumed to be the same, namely, *r_+_ = r_−_ = r* [37,42,43,45]. Substituting Equations (3)–(7) into Equations (1) and (2), we have:

(8) $$\frac{d^{2}\psi_{1}}{dy^{2}}=\frac{2zen_{0}}{\varepsilon_{1}\varepsilon_{0}}\sinh(\frac{ez}{k_{b}T_{av}}\psi_{1}),0\leq y\leq H-d$$

(9) $$\frac{d^{2}\psi_{2}}{dy^{2}}=\frac{2zen_{0}}{\varepsilon_{2}\varepsilon_{0}}\sinh(\frac{ez}{k_{b}T_{av}}\psi_{2})\exp(-\frac{\Delta W}{k_{b}T_{av}})-\frac{ZeN}{\varepsilon_{2}\varepsilon_{0}},H-d\leq y\leq H$$

The equations are named Poisson–Boltzmann (P-B) equations. The Debye–Hückel linearization is used by approximating *sinh* (*ezψ/k_b_T_av_*) to *ezψ/k_b_T_av_* so as to obtain analytical solutions. Hence, Equations (4) and (5) are simplified as:

(10) $$\frac{d^{2}\psi_{1}}{dy^{2}}=\frac{\psi_{1}}{\lambda^{2}},0\leq y\leq H-d$$

(11) $$\begin{array}{c} \frac{d^{2}\psi_{2}}{dy^{2}}=\frac{\psi_{2}}{\varepsilon \lambda^{2}}\exp(-\frac{\Delta W}{k_{b}T_{av}})-\frac{ZeN}{\varepsilon_{2}\varepsilon_{0}},H-d\leq y\leq H\\ \mathrm{with}\;\lambda =\sqrt{\frac{\varepsilon_{1}\varepsilon_{0}k_{b}T_{av}}{2e^{2}z^{2}n_{0}}},\varepsilon =\frac{\varepsilon_{2}}{\varepsilon_{1}} \end{array}$$

where *λ* represents the EDL thickness and *ε* is the permittivity ratio of the PEL to the electrolyte solution layer. Moreover, the following boundary conditions are necessary:

(12) $${\frac{d\psi_{1}}{dy}|}_{y=0}=0$$

(13) $${\psi_{1}|}_{y=H-d}={\psi_{2}|}_{y=H-d}$$

(14) $$\varepsilon_{0}\varepsilon_{1}{\frac{d\psi_{1}}{dy}|}_{y=H-d}=\varepsilon_{0}\varepsilon_{2}{\frac{d\psi_{2}}{dy}|}_{y=H-d}$$

(15) $${\frac{d\psi_{2}}{dy}|}_{y=H}=-\frac{\omega }{\varepsilon_{2}\varepsilon_{0}}$$

in which *ω* is the constant surface charge density on the slip walls. At the PEL–electrolyte interface, Equations (12) and (13) as well as (14) stand for the symmetry and continuity conditions, respectively. Equation (15) is the Gauss boundary condition on the channel walls. Here, we introduce the following nondimensional variables:

(16) $$y^{*}=\frac{y}{H},\;d^{*}=\frac{d}{H},\;(\psi_{1}{}^{*},\psi_{2}{}^{*})=\frac{\left(\psi_{1},\;\psi_{2}\right)}{k_{b}T_{av}/ez},\;\lambda^{*}=\frac{\lambda }{H},\;\Delta W^{*}=\frac{\Delta W}{k_{b}T_{av}}$$

Equations (10)–(15) are given as in nondimensional forms:

(17) $$\frac{d^{2}\psi_{1}{}^{*}}{dy^{*}{}^{2}}=\frac{\psi_{1}{}^{*}}{\lambda^{*}{}^{2}},0\leq y^{*}\leq 1-d^{*}$$

(18) $$\begin{array}{c} \frac{d^{2}\psi_{2}{}^{*}}{dy^{2}{}^{*}}=\frac{1}{\varepsilon }(\frac{w^{*}\psi_{2}{}^{*}}{\lambda^{*}{}^{2}}-\frac{1}{\lambda^{*}{{}_{FCL}}^{2}}),1-d^{*}\leq y^{*}\leq 1\\ \mathrm{with}\;w^{*}=\exp(-\Delta W^{*}),\lambda^{*}{}_{FCL}=\frac{\lambda_{FCL}}{H},\lambda_{FCL}=\sqrt{\frac{\varepsilon_{1}\varepsilon_{0}k_{b}T_{av}}{ze^{2}NZ}} \end{array}$$

where *λ_FCL_* is the equivalent EDL thickness inside the PEL.

(19) $$\begin{array}{c} {\frac{d\psi_{1}{}^{*}}{dy^{*}}|}_{y^{*}=0}=0,\\ {\psi_{1}{}^{*}|}_{y^{*}=1-d^{*}}={\psi_{2}{}^{*}|}_{y^{*}=1-d^{*}},\\ {\frac{d\psi_{1}{}^{*}}{dy^{*}}|}_{y^{*}=1-d^{*}}=\varepsilon {\frac{d\psi_{2}{}^{*}}{dy^{*}}|}_{y^{*}=1-d^{*}},\\ {\frac{d\psi_{2}{}^{*}}{dy^{*}}|}_{y^{*}=1}=-\frac{\Omega }{\varepsilon } \end{array}$$

where Ω *= Hezω/*(*k_b_T_av_ε*_1_*ε*_0_) is the dimensionless surface charge density on the walls. The general solutions of Equations (17) and (18) are expressed as:

(20) $$\psi_{1}{}^{*}(y^{*})=C_{1}\cosh(\frac{y^{*}}{\lambda^{*}}),0\leq y^{*}\leq 1-d^{*}$$

(21) $$\begin{array}{c} \psi_{2}{}^{*}(y^{*})=C_{2}\cosh(ay^{*})+C_{3}\sinh(ay^{*})+sg,1-d^{*}\leq y^{*}\leq 1\\ \mathrm{with}\;a=\frac{1}{\lambda^{*}}\sqrt{\frac{w^{*}}{\varepsilon }} \end{array}$$

Utilizing the boundary condition (19), these constants are obtained as:

(22) $$\begin{array}{c} C_{1}=\frac{C_{2}\cosh(a(1-d^{*}))+C_{3}\sinh(a(1-d^{*}))+sg}{\cosh(\frac{1-d^{*}}{\lambda^{*}})},sg={\left(\frac{\lambda^{*}}{\lambda^{*}{}_{FCL}}\right)}^{2}\frac{1}{w^{*}},\\ C_{2}=-\frac{1}{O_{1}}\left[O_{2}\Omega +sg\sinh(\frac{1-d^{*}}{\lambda^{*}})\cosh(a)\right],C_{3}=-[\frac{\Omega }{a\varepsilon \cosh(a)}+C_{2}\tanh(a)],\\ O_{1}=a\lambda^{*}\varepsilon \sinh(ad^{*})\cosh(\frac{1-d^{*}}{\lambda^{*}})+\sinh(\frac{1-d^{*}}{\lambda^{*}})\cosh(ad^{*}),\\ O_{2}=\lambda^{*}\cosh(a(1-d^{*}))\cosh(\frac{1-d^{*}}{\lambda^{*}})-\frac{1}{a\varepsilon }\sinh(\frac{1-d^{*}}{\lambda^{*}})\sinh(a(1-d^{*})) \end{array}$$

### 2.2. Fluid Velocity

For a steady, fully developed flow, the velocity fields of the thruster can be obtained in the soft nanochannel. We only take into account pure electroosmotic flows without the pressure influence in the thruster [17,18,35,45,50]. For inertia-free and unidirectional flow situations, the electrostatic driving force only balances with the viscous force in the momentum equation. Besides, the fully hydrated, dense PEL, which is grafted on the two solid walls, is modeled using Brinkman formalism in the porous media and soft electrokinetics literature [45,51]. The modified Naiver–Stokes (N-S)/Brinkman equations are represented as:

(23) $$\mu_{1}\frac{d^{2}u_{1}}{dy^{2}}+\rho_{e1}E_{x}=0,0\leq y\leq H-d$$

(24) $$\mu_{2}\frac{d^{2}u_{2}}{dy^{2}}+\rho_{e}{}_{2}E_{x}-\mu_{c}u_{2}=0,h-d\leq y\leq H$$

where *u*_1_ and *u*_2_ are axial velocities of the two layers, and *μ_c_* stands for drag coefficient of the dense PEL. *μ*_1_ and *μ*_2_ represent the dynamic viscosities within the electrolyte and PEL regions, which are considered to be different in the study. The above equations are relevant to the following boundary conditions:

(25) $${\frac{du_{1}}{dy}|}_{y=0}=0$$

(26) $${u_{1}|}_{y=H-d}={u_{2}|}_{y=H-d}$$

(27) $${\mu_{1}\frac{du_{1}}{dy}|}_{y=H-d}=\mu_{2}{\frac{du_{2}}{dy}|}_{y=H-d}$$

(28) $${{u_{2}|}_{y=H}=-\gamma \frac{du_{2}}{dy}|}_{y=H}$$

Equation (28) stands for the Naiver slip boundary condition on the walls with the slip length, *γ*. At the PEL–electrolyte interface, Equations (25)–(27) are the symmetric, continuous, and stress balance conditions, respectively. Here, we introduce some nondimensional variates:

(29) $$(u_{1}{}^{*},u_{2}{}^{*})=\frac{\left(u_{1},u_{2}\right)}{u_{HS}},u_{HS}=\frac{\varepsilon_{1}\varepsilon_{0}k_{b}T_{av}E_{x}}{ez\mu_{1}},\gamma^{*}=\frac{\gamma }{H},\mu =\frac{\mu_{2}}{\mu_{1}}$$

in which *u_HS_* is the Helmholtz–Smoluchowski electroosmotic velocity [52,53]. Dimensionless forms of Equations (23) and (24) are expressed as:

(30) $$\frac{d^{2}u_{1}{}^{*}}{dy^{*}{}^{2}}-\frac{\psi_{1}{}^{*}}{\lambda^{*}{}^{2}}=0,0\leq y^{*}\leq 1-d^{*}$$

(31) $$\begin{array}{c} \frac{d^{2}u_{2}{}^{*}}{dy^{2}{}^{*}}-\frac{w^{*}}{\mu }\frac{\psi_{2}{}^{*}}{\lambda^{*}{}^{2}}-\frac{\alpha^{2}}{\mu }u_{2}=0,1-d^{*}\leq y^{*}\leq 1\\ \mathrm{with}\;\alpha =H\sqrt{\frac{\mu_{c}}{\mu_{1}}} \end{array}$$

where *α* is the PEL drag parameter. The dimensionless forms of Equations (25)–(28) are:

(32) $$\begin{array}{c} {\frac{du_{1}{}^{*}}{dy^{*}}|}_{y^{*}=0}=0,\\ {u_{1}{}^{*}|}_{y^{*}=1-d^{*}}={u_{2}{}^{*}|}_{y^{*}=1-d^{*}},\\ {\frac{du_{1}{}^{*}}{dy^{*}}|}_{y^{*}=1-d^{*}}=\mu {\frac{du_{2}{}^{*}}{dy^{*}}|}_{y^{*}=1-d^{*}},\\ {{u_{2}{}^{*}|}_{y^{*}=1}=-\gamma^{*}\frac{du_{2}{}^{*}}{dy^{*}}|}_{y^{*}=1} \end{array}$$

Therefore, the general solutions of Equations (30) and (31) are obtained as:

(33) $$u_{1}{}^{*}(y^{*})=C_{1}\cosh(\frac{y^{*}}{\lambda^{*}})+C_{4},0\leq y^{*}\leq 1-d^{*}$$

(34) $$\begin{array}{c} u_{2}{}^{*}(y^{*})=C_{5}\cosh(by^{*})+C_{6}\sinh(by^{*})+D\cosh(ay^{*})+E\sinh(ay^{*})+F,\\ 1-d^{*}\leq y^{*}\leq 1,\\ \mathrm{with}\;b=\alpha \sqrt{\frac{1}{\mu }} \end{array}$$

where these constants are calculated as follows:

(35) $$\begin{array}{c} C_{4}=C_{5}\cosh(b-bd^{*})+C_{6}\sinh(b-bd^{*})-C_{1}\cosh(\frac{1-d^{*}}{\lambda^{*}})+D\cosh(a-ad^{*})+E\sinh(a-ad^{*})+F,\\ C_{5}=\frac{M-GQ}{1-Q\tanh(b-bd^{*})},C_{6}=-C_{5}\tanh(b-bd^{*})+G,Q=\frac{\sinh(b)+\gamma^{*}b\cosh(b)}{\cosh(b)+\gamma^{*}b\sinh(b)},\\ G=\frac{C_{1}\sinh(\frac{1-d^{*}}{\lambda^{*}})-\mu \lambda^{*}a[D\sinh(a-ad^{*})+E\cosh(a-ad^{*})]}{\mu \lambda^{*}b\cosh(b-bd^{*})},\\ D=\frac{w^{*}\varepsilon C_{2}}{\mu w^{*}-\alpha^{2}\lambda^{*}{}^{2}\varepsilon },E=\frac{w^{*}\varepsilon C_{3}}{\mu w^{*}-\alpha^{2}\lambda^{*}{}^{2}\varepsilon },F=-\frac{1}{\alpha^{2}\lambda^{*}{{}_{FCL}}^{2}},\\ M=-\frac{(D+\gamma^{*}aE)\cosh(a)+(E+\gamma^{*}aD)\sinh(a)+F}{\cosh(b)+\gamma^{*}b\sinh(b)} \end{array}$$

## 3. Thruster Performance Analysis

Performances of the electroosmotic thruster can be investigated after obtaining the fluid electric potential and velocity through the nanochannels with the dense PEL, which are thruster-specific impulse, *I_sp_*, generated thrust, *Th*, efficiency, *η_t_,* and thrust-to-power ratio, *ζ* = *Th/P_in_*.

### 3.1. Specific Impulse

Thruster-specific impulse, *I_sp_*, is defined as the propellant exhaust velocity divided by the gravitational acceleration constant [54]. In the electrolyte solution layer, the exhaust velocity is viewed as the average flow speed [17,18,35]. In dense PEL, the velocity is the superficial averaged velocity in the research [51]. Therefore, the specific impulse is given as:

(36) $$I_{sp}=\frac{1}{g_{0}H}\int_{0}^{H}u(y)dy=\frac{1}{g_{0}H}\left[\int_{0}^{H-d}u_{1}(y)dy+\int_{H-d}^{H}u_{2}(y)dy\right]$$

(37) $$\mathrm{or}\;I_{sp}{}^{*}=\int_{0}^{1-d*}u_{1}{}^{*}(y^{*})dy^{*}+\int_{1-d^{*}}^{1}u_{2}{}^{*}(y^{*})dy^{*}$$

in which I_sp_^*^ = I_sp_/(*u_HS_/g*_0_) is the nondimensional specific impulse, with *g*_0_ being the gravitational constant at sea level [54,55]. We first introduce two new functions *S*(*n*) and *C*(*n*) for convenience:

(38) $$S(n)={\frac{1}{n}\sinh(ny^{*})|}_{y^{*}=1-d^{*}}^{y^{*}=1}=\frac{1}{n}\left\{\sinh(n)-\sinh\left[n(1-d^{*})\right]\right\}$$

(39) $$\mathrm{and}\;C(n)={\frac{1}{n}\cosh(ny^{*})|}_{y^{*}=1-d^{*}}^{y^{*}=1}=\frac{1}{n}\left\{\cosh(n)-\cosh\left[n(1-d^{*})\right]\right\}$$

Substituting Equations (33) and (34) into Equation (37), the nondimensional specific impulse is written as:

(40) $$I_{sp}{}^{*}=\lambda^{*}C_{1}\sinh(\frac{1-d^{*}}{\lambda^{*}})+C_{4}+(F-C_{4})d^{*}+C_{5}S(b)+C_{6}C(b)+DS(a)+EC(a)$$

### 3.2. Thrust

Thrust of the electrolyte propellant is achieved via transforming the input electrical power into total kinetic power. The thrust is defined as the mass flow rate of fluid multiplied by the mean flow speed/superficial averaged velocity in the channel, namely *Th =* *ṁv_m_*. The mass flow rate, *ṁ*, is expressed as *ṁ* = *ρv_m_HW,* with the fluid density, *ρ*. Hence, the expression of thrust is acquired by integrating the differential thrust over the cross-sectional area of the channel:

(41) $$Th=2W\int_{0}^{H}\rho u^{2}(y)dy=2W\left[\int_{0}^{H-d}\rho_{1}{u_{1}}^{2}(y)dy+\int_{H-d}^{H}\rho_{2}{u_{2}}^{2}(y)dy\right],$$

We consider that the densities of the electrolyte solution layer, *ρ*_1_, and PEL, *ρ*_2_, are different owing to the ion partitioning and hydration effects in the soft channel. The nondimensional thrust is obtained as:

(42) $$Th^{*}=\int_{0}^{1-d^{*}}u_{1}{{}^{*}}^{2}(y^{*})dy^{*}+\int_{1-d^{*}}^{1}\rho_{r}u_{2}{{}^{*}}^{2}(y^{*})dy^{*}=Th_{1}{}^{*}+Th_{2}{}^{*},$$

(43) $$\begin{array}{c} \mathrm{with}\begin{array}{c} {Th_{1}}^{*}=\frac{C_{1}{}^{2}\lambda^{*}}{4}\sinh\left[\frac{2(1-d^{*})}{\lambda^{*}}\right]+\\ 2\lambda^{*}C_{4}C_{1}\sinh(\frac{1-d^{*}}{\lambda^{*}})+(1-d^{*})(\frac{C_{1}{}^{2}}{2}+C_{4}{}^{2}), \end{array}\\ Th_{2}{}^{*}=\rho_{r}\{\frac{1}{2}(C_{5}{}^{2}+C_{6}{}^{2})S(2b)+\frac{1}{2}(D^{2}+E^{2})S(2a)+(C_{5}D+C_{6}E)S(a+b)+\\ (C_{5}D-C_{6}E)S(b-a)+2C_{5}FS(b)+2DFS(a)+C_{5}C_{6}C(2b)+\\ \;(C_{5}E+C_{6}D)C(b+a)+(C_{5}E-C_{6}D)C(a-b)+2C_{6}FC(b)+\\ \;2EFC(a)+DEC(2a)+[\frac{1}{2}(C_{5}{}^{2}-C_{6}{}^{2}+D^{2}-E^{2})+F^{2}]d^{*}\} \end{array}$$

where Th^*^ = Th/(2*WHρ_1_u_HS_*^2^) and the density ratio *ρ_r_* = *ρ*_2_/*ρ*_1_.

### 3.3. Efficiency

The EOT total power input, *P_in_*, is calculated by investigating the total energy requirement in soft nanochannels with the dense PEL. For the pure electroosmotic-driven fluid, a part of *P_in_* provided by the power processing unit (PPU) is converted into the propellant kinetic energy, *K,* to maintain continuous operation of the thruster, meanwhile the other part of *P_in_* is consumed by other mechanisms. These mechanisms include the Joule heating effect, *P_j_*, viscous dissipation, *P_v_*, and sliding frictional heating, *P_f_*, on the walls. Two dissipation mechanisms, *P_j_* and *P_v_*, are well-understood and thought of in electroosmotic systems. Since the slip phenomena of the superficial averaged velocity is considered in our present analysis, frictional heating should be incorporated in the amount of the energy dissipated due to the effect of wall sliding friction between the electrolyte solution and the pore’s boundaries [18,35,56]. When the high-density polyelectrolyte layer is taken into account, these thruster energies are also affected by the ion partitioning, different permittivity, slip parameter, and so on. Hence, the total energy input is acquired as:

(44) $$P_{in}=K+P_{j}+P_{v}+P_{f}$$

(45) $$\mathrm{with}\;K=\int_{A_{out}}\frac{1}{2}\rho u^{3}dA_{out}=\int_{A_{out1}}\frac{1}{2}\rho_{1}u_{1}{}^{3}dA_{out}+\int_{A_{out2}}\frac{1}{2}\rho_{2}u_{2}{}^{3}dA_{out}$$

(46) $$P_{j}=\int_{V}\sigma E_{x}{}^{2}dV=\int_{V_{1}}\sigma_{1}E_{x}{}^{2}dV+\int_{V_{2}}\sigma_{2}E_{x}{}^{2}dV$$

(47) $$P_{v}=\int_{V}\mu {\left(\frac{du}{dy}\right)}^{2}dV=\int_{V_{1}}\mu_{1}{\left(\frac{du_{1}}{dy}\right)}^{2}dV+\int_{V_{2}}\mu_{2}{\left(\frac{du_{2}}{dy}\right)}^{2}dV$$

(48) $$P_{f}=\int_{A_{wall}}\mu_{2}u_{2}(y=H)\frac{du_{2}(y=H)}{dy}dA_{wall}$$

where *A_out_* stands for the cross-sectional area of the nanochannel, *V* is the total nanochannel volume, and *A_wall_* is the surface area of the wall. Subscripts 1 and 2 represent parameters of the electrolyte solution layer and PEL, respectively. *σ*_1_ and *σ_2_* are the fluid electrical conductivity for the two layers [57]:

(49) $$\sigma_{i}=\sigma_{0}\cosh(\frac{ez}{k_{b}T_{av}}\psi_{i})≅\sigma_{0}\left[1+\frac{1}{2}{\left(\frac{ez}{k_{b}T_{av}}\psi_{i}\right)}^{2}\right],i=1,2$$

where *σ*_0_ is the electrical conductivity of the neutral liquid. Taking advantage of the fluid electric potential and velocity distributions, we acquire nondimensional expressions of the mentioned energy items:

(50) $$K^{*}=\int_{0}^{1-d^{*}}\frac{1}{2}u_{1}{{}^{*}}^{3}dy^{*}+\int_{1-d^{*}}^{1}\frac{1}{2}\rho_{r}u_{2}{{}^{*}}^{3}dy^{*}=K_{1}{}^{*}+K_{2}{}^{*}$$

(51) $$P_{j}{}^{*}=\beta \left[\int_{0}^{1-d^{*}}(1+\frac{1}{2}\psi_{1}{{}^{*}}^{2})dy^{*}+\int_{1-d^{*}}^{1}(1+\frac{1}{2}\psi_{2}{{}^{*}}^{2})dy^{*}\right]=\beta (J_{1}{}^{*}+J_{2}{}^{*})$$

(52) $$P_{v}{}^{*}=\delta \left[\int_{0}^{1-d^{*}}{\left(\frac{du_{1}{}^{*}}{dy^{*}}\right)}^{2}dy^{*}+\int_{1-d^{*}}^{1}\mu {\left(\frac{du_{2}{}^{*}}{dy^{*}}\right)}^{2}dy^{*}\right]=\delta (\Phi_{1}{}^{*}+\Phi_{2}{}^{*})$$

(53) $$P_{f}{}^{*}=\delta \left[\mu u_{2}{}^{*}(y^{*}=1)\frac{du_{2}{}^{*}(y^{*}=1)}{dy^{*}}\right]=\delta P_{f_{2}}{}^{*}$$

(54) $$P_{in}{}^{*}=K_{1}{}^{*}+K_{2}{}^{*}+\beta (J_{1}{}^{*}+J_{2}{}^{*})+\delta (\Phi_{1}{}^{*}+\Phi_{2}{}^{*}+P_{f_{2}}{}^{*})$$

where [P_in_^*^, K^*^, P_j_^*^, P_v_^*^, P_f_^*^] = [P_in_, K, P_j_, P_v_, P_f_]/(2*ρ_1_HWu_HS_*^3^) are the nondimensional energy items of thrusters. Some expressions in Equations (50)–(53) are shown in the Appendix A. The parameters *β* and *δ,* which characterize the Joule heating effect and viscous frictional heating, are written as:

(55) $$\beta =\frac{L\sigma_{0}E_{x}{}^{2}}{\rho_{1}u_{HS}{}^{3}},\delta =\frac{L\mu_{1}}{H^{2}\rho_{1}u_{HS}}$$

Therefore, thruster efficiency, *η*_t_, is acquired, which is the kinetic energy divided by the total input power [54,55]:

(56) $$\eta_{t}=\frac{K}{P_{in}}=\frac{K^{*}}{P_{in}{}^{*}}=\frac{K_{1}{}^{*}+K_{2}{}^{*}}{K_{1}{}^{*}+K_{2}{}^{*}+\beta (J_{1}{}^{*}+J_{2}{}^{*})+\delta (\Phi_{1}{}^{*}+\Phi_{2}{}^{*}+P_{f_{2}}{}^{*})}$$

### 3.4. Thrust-to-Power Ratio

The thrust-to-power ratio has to be studied owing to its influence on all kinds of space mission peculiarities, such as duration of thrust, payload capability, and system cost. The dimensionless form of *ζ* *= Th/P_in_* is expressed easily as:

(57) $$\zeta^{*}=u_{HS}\zeta =\frac{Th^{*}}{P_{in}{}^{*}}=\frac{Th_{1}{}^{*}+Th_{2}{}^{*}}{K_{1}{}^{*}+K_{2}{}^{*}+\beta (J_{1}{}^{*}+J_{2}{}^{*})+\delta (\Phi_{1}{}^{*}+\Phi_{2}{}^{*}+P_{f_{2}}{}^{*})}$$

It is worth noting that *ζ* *= Th/P_in_* is in connection with the thruster efficiency and flow velocity [54], i.e.,

(58) $$\zeta =\frac{Th}{P_{in}}\;\sim \;\frac{\rho HWv_{m}{}^{2}}{K/\eta_{t}}=\eta_{t}\frac{\rho HWv_{m}{}^{2}}{\rho HWv_{m}{}^{3}/2}=\frac{2\eta_{t}}{v_{m}}$$

## 4. Results and Discussion

In the following, we shall chiefly focus on influences of the walls with the dense PEL material on the thruster performances to achieve results closer to reality and compare the present works with the previous paper [35] to provide visualized changes or improvements. Moreover, analyses about effects of various energy items are introduced, which can be illustrated by considering some correlative parameters, such as the permittivity ratio, slip length, and viscosity ratio, for the electrolyte layer and PEL. For a demonstrative case, we consider a 1:1 symmetric electrolyte solution such as KCL, whose effective ionic radius is 3.3 × 10^−10^ m. The main working fluid is assumed to be water at *T* = 300 K and the value of the applied axial electric field is set as 5 × 10^8^ V/m. Geometric scales of the thruster and material features of the working fluid are treated the same as in the previous paper [35] under the steady, fully developed condition. Moreover, in soft nanochannels at high grafting densities, the relative permittivity of the PEL, *ε*_2_, is 52.8–78 [58,59], and the viscosity ratio of liquid inside and outside the PEL layer, *μ,* ranges from 1 to 7 [41]. Therefore, all of the expressions for the thruster velocity and performances are presented by the MATLAB programming software.

**Figure 2:**
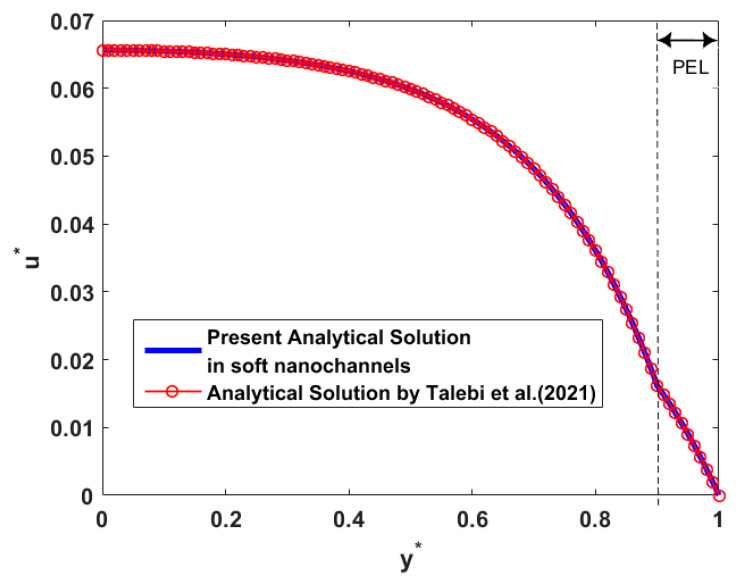
Comparison of the variations of the dimensionless velocity u* with y* in the study of Talebi et al. [45] and in the present research by setting parameters d * = 0.1, *α* = 1, γ * = 0, Ω = 0, λ * = 0.2, λ *_FCL_/λ * = 2.5, *ε* = 1, and *μ* = 2.

To verify the correctness of the current result about electroosmotic velocity in the thruster, we first compared with the previous work investigated by Talebi et al. [45]. The comparison of the dimensionless results is presented in Figure 2, because the nondimensional solutions in the study of Talebi et al. [45] are discussed and the scale of the channel model is different in the two studies. In soft channels with the dense polyelectrolyte layer (PEL), our result is in accordance with the velocity distribution in the research of Talebi et al. [45] after converting the wall surface charge density condition and Naiver slip boundary into no surface charge density and no slip boundary conditions on the walls, as shown in Figure 2. Besides, the discontinuity of flow velocity at the electrolyte–PEL interface is obvious owing to the differences of the dielectric permittivity and viscosity for the two layers. The stress balance condition in Equation (27) further explains the discontinuity of the velocity on the electrolyte–PEL interface utilizing the different viscosities for the two layers.

**Figure 3:**
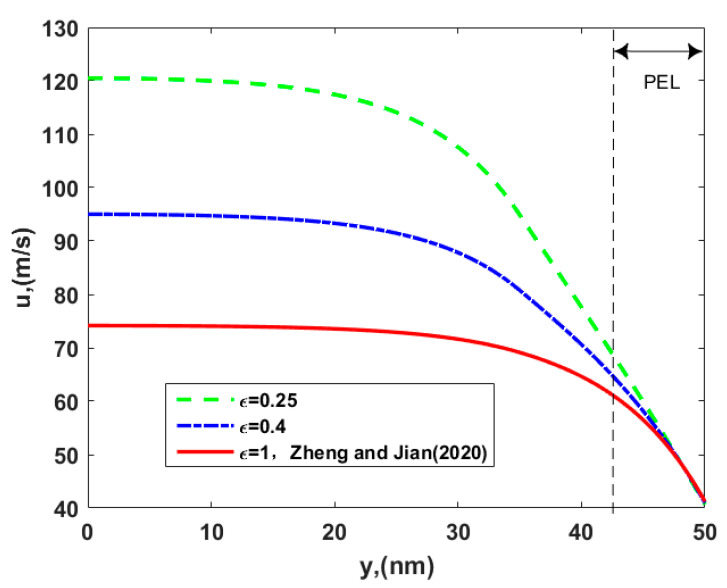
The velocity distribution of electroosmotic thrusters in the soft nanochannel for the different permittivity ratio of layer II to layer I, *ε*, with *d* = 15 nm, *γ* = 40 nm, *μ_c_* = 0.34 Pa s/m^2^, *ω* = −6.5 mC/m^2^, *λ* = 7.5 nm, λ*_FCL_/λ* = 2, and *μ* = 1.

In Figure 3, the velocity profile is described for the different permittivity ratio, *ε,* of the two layers. The electroosmotic velocity increases as the permittivity ratio decreases. The reduction means that the permittivity of PEL becomes small, which is the reason why ions in the channel prefer to stay within the electrolyte solution layer instead of the dense PEL, i.e., the smaller the permittivity, the smaller the ability to bind the charge. The ion partitioning leads to an enhancement of the ionic concentration outside the PEL, where polyelectrolyte molecules are absent and there is no resistive body force (Stokes retarding force) on fluid particles, so an obvious growth in the velocity appears as the permittivity ratio decreases. Hence, the present velocity is larger than that of Zheng and Jian [35] with relevant parameters *ε = μ* = 1 in Figure 3.

**Figure 4:**
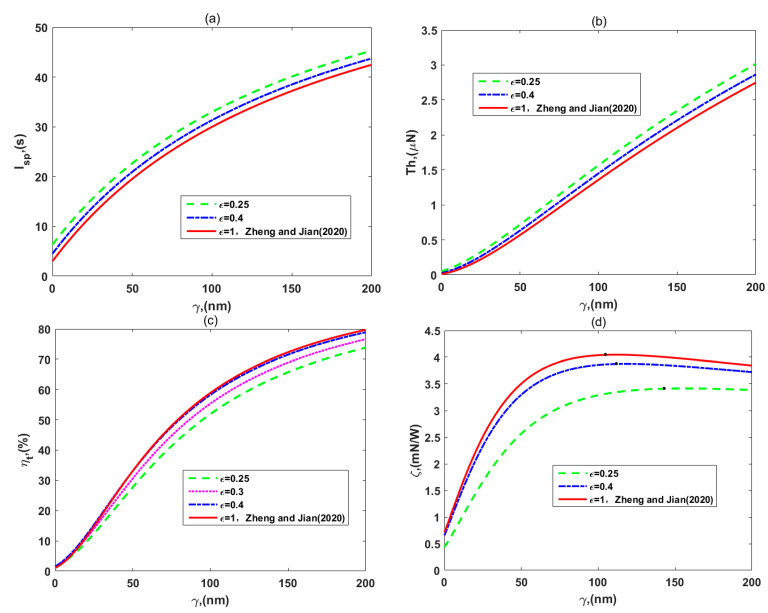
The variations of (**a**) specific impulse, *I_sp_*, (**b**) thrust, *Th*, (**c**) efficiency, *η_t_*, and (**d**) thrust-to-power ratio, *ζ,* for the different permittivity ratios for the two layers, *ε*, with *d* = 15 nm, *μ_c_* = 0.34 Pa s/m^2^, *ω* = −6.5 mC/m^2^, *λ* = 7.5 nm, *λ_FCL_/λ* = 2, *μ* = 1, and *ρ_r_* = 3.

Figure 4 presents the variations of the (a) specific impulse, *I_sp_*, (b) thrust, *Th*, (c) thruster efficiency, *η_t_*, and (d) thrust-to-power ratio, *ζ,* which are plotted for the different permittivity ratio of layer II to layer I, *ε*. Figure 4a, b show that the thruster-specific impulse and thrust increase as the values of the permittivity ratio decrease for fixing a slip length. These results agree with those of Figure 3, namely that the ion partitioning effect enhances the EOF velocity. This is due to the fact that *I_sp_* and *Th* are respectively proportional to the velocity and to the square of the velocity according to Equations (36) and (41). Physically speaking, the higher the impulse generated by the unit amount of propellant, namely the specific impulse, the greater the velocity increment that the propellant provides under the same conditions, and the greater the thrust.

In Figure 4c,d, the thruster efficiency and thrust-to-power ratio reduce as the permittivity ratio decreases, which is different from the variation of the other two thruster performances. According to the expression (56) of the efficiency, the denominator, total power input, grows faster than the numerator, the propellant kinetic energy, when *ε* reduces. The reason is that the viscous dissipation and sliding frictional heating increase as the velocity increment becomes large, while the Joule heating effect is not influenced by it. In other words, although the ion partitioning effect leads to an increase in the velocity increment, the dissipation of the system has to be growing in terms of Equations (46)–(48). Additionally, we can easily explain the variation of the thrust-to-power ratio in terms of Equation (58), where the numerator, thruster efficiency, decreases while the denominator, flow velocity, increases, when *ε* reduces. It is interesting that the influence of the ion partitioning on thruster efficiency and peak values of the thrust-to-power ratio are not noticeable when the permittivity ratio is close to 1. Moreover, in each part of Figure 4, the changes or improvements of thruster performances are simultaneously presented, from the weak PEL investigated by Zheng and Jian [35] to the dense PEL. The specific impulse and thrust of nano-thrusters in soft channels with the dense PEL are larger than those with weak grafting density of the polyelectrolyte layer via setting parameters *ε = μ* = 1. Therefore, if the dense PEL in soft nanochannels is considered, the electroosmotic thruster can offer more power and impulse to complete space missions.

**Figure 5:**
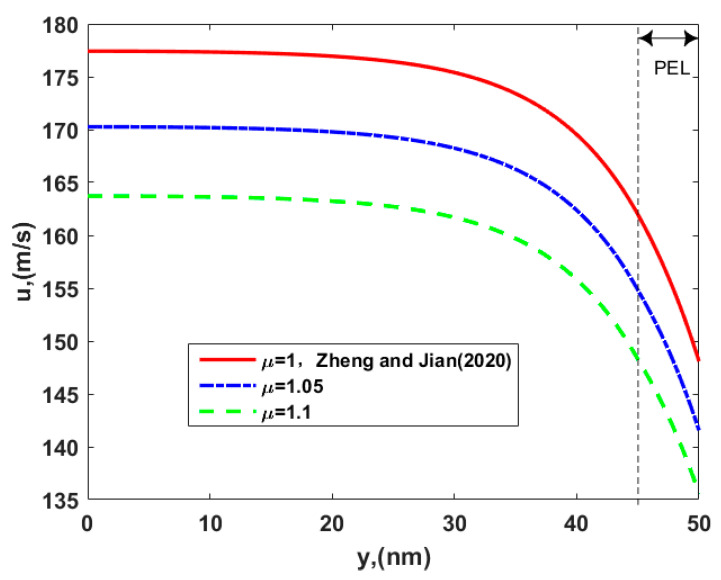
The velocity distribution of the electroosmotic thruster in the soft nanochannel for the different viscosity ratio of layer II to layer I, *μ*, with *d* = 5 nm*, γ* = 40 nm*, μ_c_* = 0.34 Pa s/m^2^*, ω* = −6.5 mC/m^2^*, λ* = 7.5 nm*, λ_FCL_/λ* = 2, and *ε* = 1.

Figure 5 presents the influence of the viscosity ratio of layer II to layer I, *μ,* on the electroosmotic velocity. The increase of *μ* generates more resistance of the fluid against flowing in the entire channel, leading to a decrease of the flow speed. Since a larger viscosity ratio means a larger viscosity of the PEL for a fixed *μ*_1_, the enhanced viscous force inside the PEL retards the fluid motion. This dense PEL is similar to a polymer brush with a lot of teeth, they produce some resistance to the fluid as the liquid flows through this layer. Therefore, the present velocity of thrusters is smaller than that of Zheng and Jian [35], who did not consider that the PEL was dense, as shown in Figure 5.

**Figure 6:**
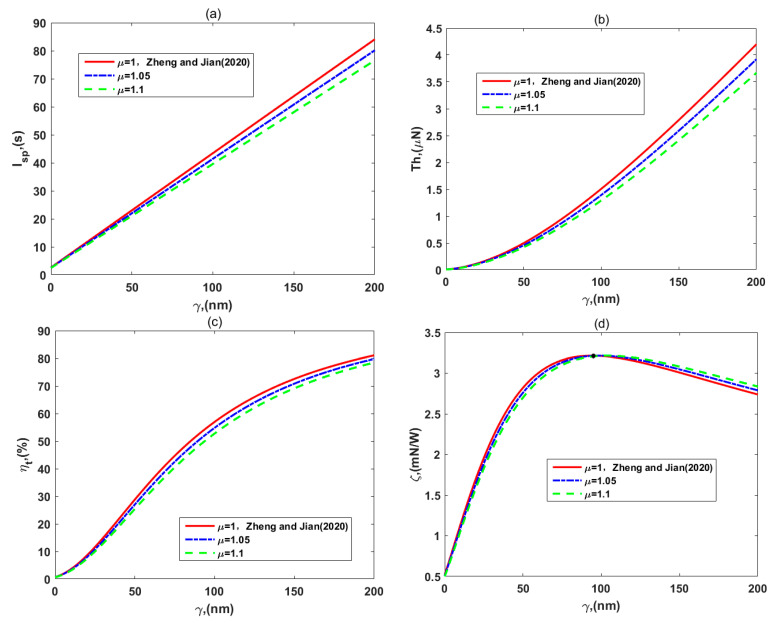
The variations of (**a**) specific impulse, *I_sp_*, (**b**) thrust, *Th*, (**c**) efficiency, *η_t_*, and (**d**) thrust-to-power ratio, *ζ,* for the different viscosity ratio of layer II to layer I, *μ*, with *d* = 5 nm*, μ_c_* = 0.34 Pa s/m^2^, *ω* = −6.5 mC/m^2^, *λ* = 7.5 nm, *λ_FCL_/λ* = 2, *ε* = 1, and *ρ_r_* = 3.

In Figure 6, we investigate the variations of the (a) specific impulse, *I_sp_*, (b) thrust, *Th*, (c) thruster efficiency, *η_t_,* and (d) thrust-to-power ratio, *ζ,* for different values of the viscosity ratio, *μ.* It is obvious in Figure 6a–c that *I_sp_*, *Th*, and *η_t_* decrease with the viscosity ratio of the two layers for a given slip length. There is a reasonable physical sense that more resistances generated by the dense PEL lead to the lower impulse, thrust, and kinetic energy that the propellant provides. In Figure 6d, the influence of the viscosity ratio, *μ,* on the thrust-to-power ratio, *ζ,* is shown. For no slip and small slip length cases, enhancement of the viscosity ratio causes a reduction of the thrust-to-power ratio because the thrust reduces faster than the total power. On the other hand, when the slip length becomes gradually large, the thrust-to-power ratio increases with the viscosity ratio, since the denominator of Equation (58) eventually reduces faster than the numerator of it does.

Specifically, peak values of the thrust-to-power ratio in Figure 6d are identical for three values of the viscosity ratio. That is a good result, where the viscosity ratio has little influence on the peak values of the thrust-to-power ratio. Simultaneously, their variations from the weak PEL to the dense PEL are shown in each part of Figure 6, and the research of Zheng and Jian [35] is marked too. As was predicted, the thruster-specific impulse, thrust, and efficiency with the dense PEL were smaller than those with weak grafting density of the polyelectrolyte layer, but the peak values of the thrust-to-power ratio did not change much. This finding is unfavorable for these performances of thrusters, so we can adjust other parameters to weaken the effect of resistances generated by the dense PEL on the flow, such as decreasing the viscosity of layer I or changing EDL thickness, and so on. Therefore, the influences of EDL thickness on the flow velocity and thruster performances are further considered in the following figures.

**Figure 7:**
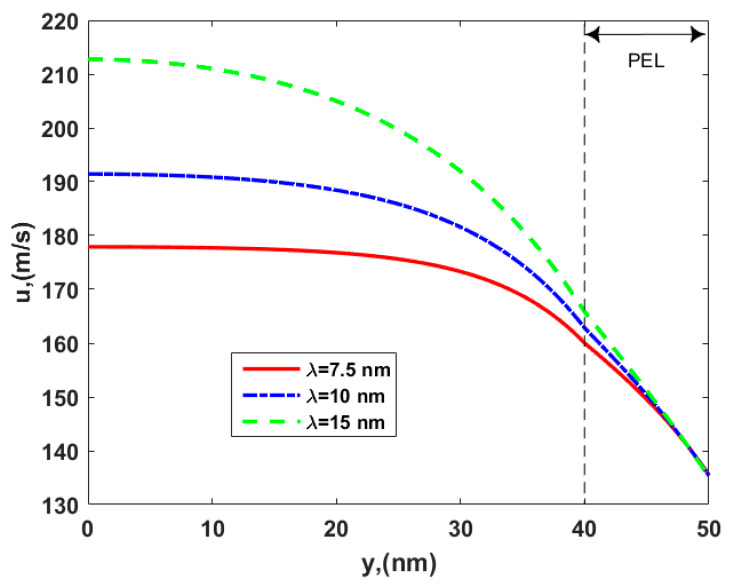
The velocity distribution of the electroosmotic thruster in the soft nanochannel for the different EDL thicknesses, *λ*, with *d* = 10 nm*, γ* = 40 nm*, μ_c_* = 0.34 Pa s/m^2^*, ω* = −6.5 mC/m^2^*, λ_FCL_* = 10 nm, *μ =* 1.25, and *ε* = 0.5.

After setting other parameters, Figure 7 shows the velocity variation of electroosmotic thrusters along with the EDL thickness, *λ,* in the soft nanochannel with dense PEL. The velocity increases with the EDL thickness since the electroosmotic body force in the EDL enhances so as to promote the fluid flow outside the PEL, where there is no Stokes retarding force. Hence, the large velocity is obtained in the channel due to the enhancement of the EDL thickness, which might be a parameter to provide some optimizations for practical designing and manufacturing of the thruster.

**Figure 8:**
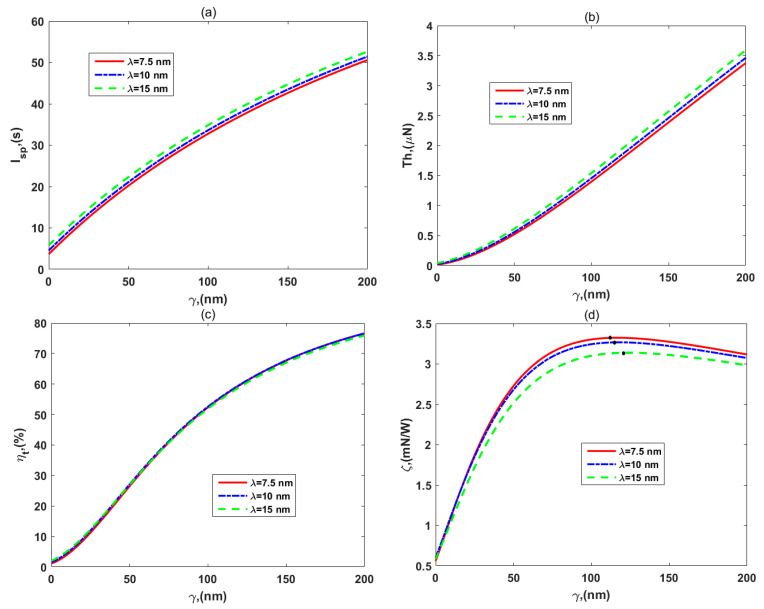
The variations of (**a**) specific impulse, *I_sp_*, (**b**) thrust, *Th*, (**c**) efficiency, *η_t_*, and (**d**) thrust-to-power ratio, *ζ,* for the different EDL thicknesses, *λ,* with *d* = 10 nm*, μ_c_* = 0.34 Pa s/m^2^, *ω* = −6.5 mC/m^2^, *λ_FCL_* = 10 nm, *μ* = 1.25, *ε* = 0.5, and *ρ_r_* = 3.

Figure 8a–d present the performance distributions of specific impulse, *I_sp_*, thrust, *Th*, thruster efficiency, *η_t_,* and thrust-to-power ratio, *ζ*, respectively, with regard to the different the EDL thicknesses, *λ.* It was found that the enhancement of EDL thickness promotes the development of thrust and specific impulse owing to the increase in the electroosmotic body force outside PEL. This means that we could improve the EDL thickness to obtain more thrust and kinetic energy for different space missions. However, the thruster efficiency is not affected, as shown in Figure 8c, since the dissipation of the system increases along with the EDL thickness. In Figure 8d, the thrust-to-power ratio decreases with the EDL thickness, which can be explained by Equation (58), again, that the denominator, flow velocity, increases with the EDL thickness, but the numerator, thruster efficiency, is immutable. In spite of that, the efficiency and the peak value of the thrust-to-power ratio are still decent and outstanding.

**Figure 9:**
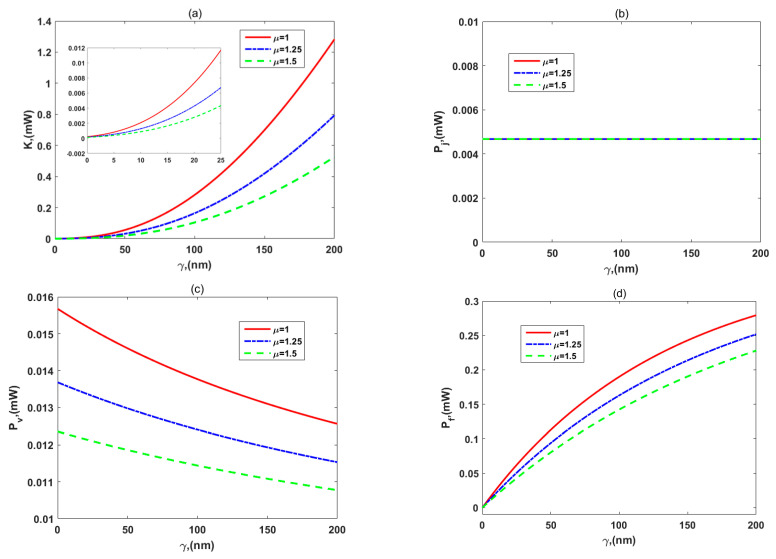
The distributions of (**a**) the propellant kinetic energy, *K*, (**b**) the Joule heating effect, *P_j_*, (**c**) viscous dissipation, *P_v_*, and (**d**) slip frictional heating, *P_f_,* on the walls for the slip length, *γ*, for the different viscosity ratio for the two layers, *μ*, with *d* = 5 nm*, μ_c_* = 0.34 Pa s/m^2^, *ω* = −6.5 mC/m^2^*, λ* = 7.5 nm*, λ_FCL_/λ* = 2, *ε* = 0.5, and *ρ_r_* = 3.

Moreover, the distributions of various energy items, including the propellant kinetic energy, *K*, the Joule heating effect, *P_j_*, viscous dissipation, *P_v_*, and slip frictional heating, *P_f_*, are investigated in Figure 9a–d, since the dense PEL also strongly alters them. The ion partitioning effect is considered by setting the value of the permittivity ratio for the two layers as *ε =* 0.5. As was expected, the kinetic energy decreased with the viscosity ratio for a given slip length in Figure 9a. Whatever the value of *μ* is, the kinetic energy increases with *γ*. The reason is that the enhancement of the slip length promotes the fluid flow so as to generate more kinetic energy. According to Equation (46), the Joule heating effect is independent of the viscosity ratio and slip length, so that three parallel lines are coincident for three different values of the viscosity ratio in Figure 9b. In other words, the Joule heating dissipation is produced by the constant external electric field and the electric potential of the two layers, while they are independent of the viscosity and slip length. In Figure 9c, d, when a slip length value is set, the viscous dissipation and slip frictional heating reduce with the viscosity ratio since the shear rate of velocity in the vicinity of the electrolyte–PEL interface plays a leading role in Equations (47) and (48). It is a fact that the decrease in specific impulse means a reduction of the velocity increment when the viscosity ratio increases, and the rate of variation in velocity has a more important influence on the system dissipation than the change of viscosity. Hence, we can reduce the dissipation items of thrusters via increasing viscosity in the dense PEL. Moreover, the frictional dissipation on the slippery walls increases with the slip length, while the viscous dissipation is the opposite.

It is noted that for no-slip and small slip length cases, the slip frictional dissipation can be neglected according to Figure 9d, and the viscous dissipation and Joule heating effect play the major roles in dissipation systems. However, the thruster efficiency is very small due to a small amount of kinetic energy as shown in Figure 9a, when there is no slip length or small slip length. As the slip length becomes gradually large, the viscous dissipation decreases, but the slip frictional dissipation increases, which means that the slip frictional heating has to be considered in the present study owing to using the slip boundary condition. Besides, we could further estimate the amount of viscous dissipation contributed by the liquid and the amount of frictional heating contributed by the PEL solid backbone by choosing and modifying the viscosity ratio and slip parameter according to Figure 9c,d. It is beneficial to the optimization in the efficiency and thrust–power ratio and the reduction of the system dissipation.

Finally, based on all parametric discussions, we designed and provided two modeled electroosmotic thrusters, EOT_1_ and EOT_2_, shown in Table 1 with full details, so as to more intuitively present improvements of thruster performances in this study.

## 5. Conclusions

In the present article, theoretical research was acquired on space electroosmotic thrusters in a soft nanochannel with a dense polyelectrolyte layer (PEL). The layer is dense, so the ion partitioning effect and different viscosities for the electrolyte solution layer and PEL were considered in this study. The linearized Poisson–Boltzmann equation and modified Naiver–Stokes/Brinkman equation were solved to obtain analytical solutions of the EDL electric potential and flow velocity under a steady, fully developed circumstance, taking the Naiver slip boundary and constant surface charge density on the walls into account. Thruster performances were further calculated, which were the thrust, specific impulse, thruster efficiency, and thrust-to-power ratio, with power consumption including the kinetic energy, Joule heating, viscous dissipation, and sliding frictional heating. The impacts of dense polyelectrolyte layers were investigated and explored to obtain results closer to the reality as well as to optimize the EOT design in soft nanochannels.

The results indicate that the ion partitioning effect improves the fluid velocity in soft nanochannels owing to an enhancement of the net ionic concentration within the electrolyte solution layer. Moreover, different viscosities for the two layers also amplified the electroosmotic flow so that the thruster performances changed. The thruster-specific impulse and thrust increased as the permittivity ratio and viscosity ratio for the two layers decreased. When the permittivity ratio was close to 1, it was interesting that the influence of the ion partitioning on thruster efficiency was not noticeable. The enhancement of EDL thickness promoted the development of specific impulse and thrust so as to obtain more kinetic energy. The variation of the viscosity ratio did not affect the peak values of the thrust-to-power ratio. Moreover, when the no-slip and small slip length cases were considered in some studies, the slip frictional dissipation could be neglected, and the amount of viscous dissipation was not very large. Compared with one of the EOTs with walls of low-density polyelectrolyte materials, the thruster efficiency in the present study was improved by about 90.40% and thrust was able to attain 0 to 20 μN, levels which conform to the industrial requirements in the TianQin space detector study [60].

It is hopeful that these new results acquired in the research could further provide a more theoretical basis for the space propulsion of electroosmotic thrusters in soft nanochannels [35]. However, the study is an analytical solution analysis and any experimental prototype might meet challenges, for instance in manufacture of a thruster with ion partitioning soft nanochannels, which is likely to affect the final performances and is worth studying in the future.

## Figures and Tables

**Table 1:** Design parameters and performance peculiarity for two EOTs in the ion partitioning soft nanochannel.

Design	Parameters								Single Emitter Performances			
	*ε*	*μ*	*λ_FCL_/λ*	*ω* (mC/m^2^)	*μ_c_* (Pa s/m^2^)	*γ* (nm)	*d* (nm)	*ρ_r_*	*I_sp_* (s)	*Th* (μN)	*ζ* (mN/W)	*η_t_* (*%*)
EOT_1_	0.75	1	0.5	−6.5	0.0034	175	10	3.5	121.4	20.84	1.53	90.40
EOT_2_	0.25	1.05	1	−6.5	0.09	175	10	3.5	70.97	6.98	2.41	82.46

## Data Availability

Not applicable.

## Appendix A. Dimensionless Expressions of the EOT Energy

The energy expressions in Equations (50)–(54) are presented as follows:

$$\begin{array}{c} K_{1}{}^{*}=\frac{1}{2}\{\sinh[\frac{3(1-d^{*})}{\lambda^{*}}]\frac{C_{1}{}^{3}\lambda^{*}}{12}+\sinh[\frac{2(1-d^{*})}{\lambda^{*}}]\frac{3C_{1}{}^{2}C_{4}\lambda^{*}}{4}+\\ \sinh(\frac{1-d^{*}}{\lambda^{*}})(\frac{3C_{1}{}^{3}\lambda^{*}}{4}+3C_{4}{}^{2}C_{1}\lambda^{*})+(1-d^{*})(C_{4}{}^{3}+\frac{3C_{1}{}^{2}C_{4}}{2})\}\\ K_{2}{}^{*}=\frac{\rho_{r}}{2}[(\frac{C_{5}{}^{3}}{4}+\frac{3C_{6}{}^{2}C_{5}}{4})S(3b)+(\frac{3C_{5}{}^{3}}{4}-\frac{3C_{6}{}^{2}C_{5}}{4}+\frac{3D^{2}C_{5}}{2}-\frac{3E^{2}C_{5}}{2}+3F^{2}C_{5})S(b)+\\ (\frac{3C_{5}{}^{2}C_{6}}{4}-\frac{3C_{6}{}^{3}}{4}+\frac{3D^{2}C_{6}}{2}-\frac{3E^{2}C_{6}}{2}+3F^{2}C_{6})C(b)+(\frac{D^{3}}{4}+\frac{3E^{2}D}{4})S(3a)+3DEFC(2a)+\\ (\frac{3D^{3}}{4}+\frac{3DC_{5}{}^{2}}{2}-\frac{3DC_{6}{}^{2}}{2}-\frac{3E^{2}D}{4}+3F^{2}D)S(a)+(\frac{E^{3}}{4}+\frac{3ED^{2}}{4})C(3a)++3C_{6}C_{5}FC(2b)+\\ (\frac{3D^{2}E}{4}-\frac{3E^{3}}{4}+\frac{3EC_{5}{}^{2}}{2}-\frac{3EC_{6}{}^{2}}{2}+3F^{2}E)C(a)+(\frac{3FD^{2}}{2}+\frac{3FC_{5}{}^{2}}{2}-\frac{3FC_{6}{}^{2}}{2}-\frac{3E^{2}F}{2}+F^{3})d^{*}\\ +(\frac{3E^{2}C_{5}}{4}+\frac{3C_{6}DE}{2}+\frac{3D^{2}C_{5}}{4})S(b+2a)+(\frac{3E^{2}C_{5}}{4}-\frac{3C_{6}DE}{2}+\frac{3D^{2}C_{5}}{4})S(b-2a)+\\ (\frac{3DC_{6}{}^{2}}{4}+\frac{3C_{6}C_{5}E}{2}+\frac{3DC_{5}{}^{2}}{4})S(2b+a)+(\frac{3DC_{6}{}^{2}}{4}-\frac{3C_{6}C_{5}E}{2}+\frac{3DC_{5}{}^{2}}{4})S(2b-a)+\\ \left(\frac{3D^{2}F}{2}+\frac{3E^{2}F}{2})S(2a)+(\frac{3EC_{6}{}^{2}}{4}+\frac{3C_{6}C_{5}D}{2}+\frac{3EC_{5}{}^{2}}{4})C(2b+a)+(3C_{5}DF-3C_{6}EF)S(b-a\right)\\ +(\frac{3EC_{5}{}^{2}}{4}-\frac{3C_{6}C_{5}D}{2}+\frac{3EC_{6}{}^{2}}{4})C(a-2b)+(\frac{3E^{2}C_{6}}{4}-\frac{3EC_{5}D}{2}+\frac{3D^{2}C_{6}}{4})C(b-2a)+\\ \left(\frac{3E^{2}C_{6}}{4}+\frac{3EC_{5}D}{2}+\frac{3D^{2}C_{6}}{4})C(b+2a)+(3C_{5}DF+3C_{6}EF)S(b+a)+(\frac{3C_{5}{}^{2}F}{2}+\frac{3C_{6}{}^{2}F}{2})S(2b\right)\\ +(3C_{5}EF+3C_{6}DF)C(b+a)+(3C_{5}EF-3C_{6}DF)C(a-b)+(\frac{C_{6}{}^{3}}{4}+\frac{3C_{5}{}^{2}C_{6}}{4})C(3b)]\\ J_{1}{}^{*}=(1+\frac{C_{1}{}^{2}}{4})(1-d^{*})+\frac{C_{1}{}^{2}\lambda^{*}}{8}\sinh[\frac{2(1-d^{*})}{\lambda^{*}}]\\ J_{2}{}^{*}=(1+\frac{C_{2}{}^{2}}{4}-\frac{C_{3}{}^{2}}{4}+\frac{sg^{2}}{2})d^{*}+(\frac{C_{2}{}^{2}}{4}+\frac{C_{3}{}^{2}}{4})S(2a)+\frac{C_{2}C_{3}}{2}C(2a)+C_{2}sgS(a)+C_{3}sgC(a)\\ P_{f_{2}}{}^{*}=\mu [C_{5}\cosh(b)+C_{6}\sinh(b)+D\cosh(a)+E\sinh(a)+F][bC_{5}\sinh(b)+\\ bC_{6}\cosh(b)+aD\sinh(a)+aE\cosh(a)]\\ \Phi_{2}{}^{*}=\mu [(\frac{b^{2}C_{5}{}^{2}}{2}+\frac{b^{2}C_{6}{}^{2}}{2})S(2b)+(\frac{b^{2}C_{6}{}^{2}}{2}-\frac{b^{2}C_{5}{}^{2}}{2}+\frac{a^{2}E^{2}}{2}-\frac{a^{2}D^{2}}{2})d^{*}+(\frac{a^{2}D^{2}}{2}+\frac{a^{2}E^{2}}{2})S(2a)\\ +b^{2}C_{5}C_{6}C(2b)+(abC_{5}D+abC_{6}E)S(b+a)+(abC_{6}E-abC_{5}D)S(b-a)+\\ \left(abC_{6}D+abC_{5}E)C(a+b)+(abC_{6}D-abC_{5}E)C(a-b)+a^{2}DEC(2a)\right]\\ \Phi_{1}{}^{*}=\frac{C_{1}{}^{2}}{2\lambda^{*}{}^{2}}[\frac{\lambda^{*}}{2}\sinh(\frac{2(1-d^{*})}{\lambda^{*}})-(1-d^{*})] \end{array}$$
